# Utility of 2-[^18^F]Fluoro-2-deoxy-D-glucose positron emission tomography/computed tomography in assessing treatment response after chemoradiotherapy for cervical cancer

**DOI:** 10.1093/jrr/rrag060

**Published:** 2026-08-06

**Authors:** Yuto Kishimoto, Motoharu Sasaki, Hideki Otsuka, Akiko Kubo, Chisato Tonoiso, Takashi Kawanaka, Takaharu Kudoh, Hitoshi Ikushima

**Affiliations:** Course of Radiological Sciences, Graduate School of Health Sciences, Tokushima University, 3-18-15 Kuramoto-cho, Tokushima, Tokushima 770-8503, Japan; Department of Therapeutic Radiology, Institute of Biomedical Sciences, Tokushima University Graduate School, 3-18-15 Kuramoto-cho, Tokushima, Tokushima 770-8503, Japan; Department of Medical Imaging/Nuclear Medicine, Institute of Biomedical Sciences, Tokushima University Graduate School, 3-18-15 Kuramoto-cho, Tokushima, Tokushima 770-8503, Japan; Department of Radiology and Radiation Oncology, Institute of Biomedical Sciences, Graduate School, Tokushima University Tokushima, 3-18-15 Kuramoto-cho, Tokushima, Tokushima 770-8503, Japan; Department of Radiation Oncology, Tokushima University Hospital, 2-50-1 Kuramoto-cho, Tokushima, Tokushima 770-8503, Japan; Department of Radiology and Radiation Oncology, Institute of Biomedical Sciences, Graduate School, Tokushima University Tokushima, 3-18-15 Kuramoto-cho, Tokushima, Tokushima 770-8503, Japan; Department of Oral Surgery, Tokushima University Graduate School of Biomedical Sciences, 3-18-15 Kuramoto-cho, Tokushima, Tokushima 770-8504, Japan; Department of Therapeutic Radiology, Institute of Biomedical Sciences, Tokushima University Graduate School, 3-18-15 Kuramoto-cho, Tokushima, Tokushima 770-8503, Japan

**Keywords:** ^18^F-FDG-PET/CT, PERCIST, radiotherapy, uterine cervical cancer

## Abstract

Cervical cancer is the fourth most common malignancy globally. As many cases are locally advanced, early prediction of local recurrence, which often occurs within 2 years of chemoradiotherapy (CCRT), is crucial. The National Comprehensive Cancer Network Guidelines recommend post-treatment 2-[^18^F]fluoro-2-deoxy-D-glucose positron emission tomography/computed tomography (^18^F-FDG-PET/CT) for response assessment, but this is not standard in Japan. We aimed to investigate the utility of ^18^F-FDG-PET/CT in assessing primary tumor response after CCRT for cervical cancer. Seventy-three patients with cervical cancer who received curative CCRT and underwent pre- and post-treatment magnetic resonance imaging and ^18^F-FDG-PET/CT were analyzed. We collected PET metrics (maximum standardized uptake value [SUV_max_], metabolic tumor volume [MTV], total lesion glycolysis [TLG]) and classified the response. Receiver operating characteristic (ROC) curve analysis was performed on the post-treatment PET data. Univariate analysis was performed to determine the prognostic factors for local control. ROC curve analysis yielded high negative predictive values for local recurrence of post-treatment MTV, SUV_max_ and TLG. Univariate analysis showed significant differences in local control for post-treatment MTV, SUV_max_, TLG and PET Response Criteria in Solid Tumors, but not for Response Evaluation Criteria in Solid Tumors (RECIST). Functional assessment using ^18^F-FDG-PET/CT after CCRT for cervical cancer is useful for complementing conventional morphological evaluation and improving the accuracy of response assessment.

## INTRODUCTION

Cervical cancer is the fourth most common malignancy among women worldwide [1], and ~10 000 women are diagnosed annually in Japan, making it the fifth most common cancer in women. Although the incidence and mortality rates of cervical cancer have recently been declining owing to the widespread adoption of screening [2], ~45% of cases diagnosed in Japan are locally advanced cervical cancer (LACC). Local recurrence is frequent in LACC, with the majority occurring within 2 years of the initial treatment [3]. Therefore, to improve prognosis, it is crucial to predict recurrence and implement preventative adjuvant therapy as early as possible.

Magnetic resonance imaging (MRI) is a widely used therapeutic evaluation tool in cervical cancer [4–6]. MRI excels at depicting soft tissues and is a useful imaging modality from staging to post-treatment follow-up. Recent reports have also suggested that MRI-guided brachytherapy improves local control rates [7]. However, when comparing tumor size measured using MRI with the pathological tumor size after surgery, although the difference in mean values was small, reports showed that a significant discrepancy existed in ~20% of individual cases, indicating that MRI does not accurately reflect the tumor in all patients [8]. This suggests that MRI primarily provides a morphological assessment, making it difficult to evaluate changes in tumor viability early after treatment.

The Response Evaluation Criteria in Solid Tumors (RECIST), which is used as a criterion for assessing the therapeutic effect of cancer, is based on morphological diagnosis using computed tomography (CT) or MRI and thus has the challenge of being unable to capture early changes in tumor viability after treatment. In contrast, 2-[^18^F]fluoro-2-deoxy-D-glucose positron emission tomography/CT (^18^F-FDG-PET/CT) reflects glucose metabolism, allowing for the early detection of changes in tumor viability, and its usefulness in assessing therapeutic effects and predicting prognosis has been reported in many cancer types [9–11]. Correspondingly, there is an international trend toward transitioning to the PET Response Criteria in Solid Tumors (PERCIST), which uses PET data for response evaluation. However, few studies have reported the usefulness of PERCIST for predicting the prognosis of cervical cancer.

Furthermore, although the National Comprehensive Cancer Network Guidelines recommend post-treatment ^18^F-FDG-PET/CT for assessing treatment responses following chemoradiotherapy (CCRT) for cervical cancer, it has not been adopted as a standard recommendation in Japan because of the lack of high-quality clinical trial data targeting the Japanese population. The usefulness of ^18^F-FDG-PET/CT based on Japanese data is expected to contribute to the early diagnosis of residual or recurrent disease in patients with cervical cancer.

This study aimed to compare the RECIST and PERCIST in assessing the treatment response of primary tumors following CCRT for cervical cancer and to investigate the utility of ^18^F-FDG-PET/CT.

## MATERIALS AND METHODS

### Overview

This study included patients with cervical cancer scheduled to receive definitive radiotherapy (RT). Pelvic MRI and whole-body ^18^F-FDG-PET/CT were performed 1 month before RT initiation. Post-treatment imaging included pelvic MRI 1 month after the completion of RT and whole-body ^18^F-FDG-PET/CT 2–3 months after the completion of RT, followed by image analysis.

In the image analysis, three different thresholds were defined to determine the optimal metabolic tumor volume (MTV) for evaluation, and their predictive accuracy was assessed. The most favorable threshold was used for the receiver operating characteristic (ROC) curve and univariate analysis. Furthermore, residual/recurrence predictive accuracy was investigated by combining various imaging parameters.

### Patient characteristics

The overall cohort consisted of 103 patients with cervical cancer who received definitive RT and underwent ^18^F-FDG-PET/CT and MRI before and after treatment at [The Authors Institution] between May 2006 and March 2018. From this cohort, 73 patients who met the PERCIST criteria were extracted for the final analysis [12]. To assess the potential impact of excluding patients based on PERCIST criteria, we compared the study cohort (*n* = 73) with the excluded group (*n* = 30). Student’s *t*-tests revealed no significant differences in age, tumor size, post-treatment MTV, post-treatment SUV_max_ or post-treatment TLG (two-tailed *P*-values: 0.224, 0.292, 0.978, 0.659 and 0.341, respectively). Additionally, Fisher’s exact tests were performed to compare local recurrence, primary tumor size (≥4 cm vs <4 cm), International Federation of Gynecology and Obstetrics (FIGO) stage (I/II vs III/IV), and histological subtype (squamous cell carcinoma (SCC) vs others). While a significant difference was observed only in FIGO stage (*P* = 0.017), no significant differences were found in local recurrence (*P* = 0.252), tumor size (*P* = 0.467), or histology (*P* = 0.342). Given that most parameters—including metabolic kinetics, clinical outcomes and tumor size—remained consistent between the two groups, we concluded that the exclusion of these patients did not introduce significant selection bias.

The details of the PERCIST criteria employed in this study are as follows:

(1) The differences in the mean standardized uptake value corrected for lean body mass (SUL_mean_) of the liver between the pre- and post-treatment PET scan were ≤0.3 SUL units and ≤20% of the greater SUL_mean_ value.
(2) The difference in the time interval between radiopharmaceutical administration and image acquisition for the pre- and post-treatment scans was ≤15 min.
(3) The time interval between radiopharmaceutical administration and image acquisition for each scan was 50–70 min.
(4) The difference in the administered dose of the radiopharmaceutical between the pre- and post-treatment scans was ≤20%.
(5) Patients fasted for a minimum of 4 h prior to radiopharmaceutical administration, and their serum glucose levels prior to administration were <200 mg/dl.
(6) The imaging equipment used for both pre- and post-treatment scans was identical.

Clinical data for all patients, including treatment details, imaging findings, age at the start of RT, metabolic response assessment, tumor pathology, FIGO stage, primary tumor size, presence and site of recurrence, and survival status, were collected from the electronic hospital information system of [The Authors Institution]. Metabolic responses were also collected from the hospital system, which were classified by the radiologist or attending physician as complete metabolic response (CMR) if post-treatment uptake on ^18^F-FDG-PET/CT images was judged to have decreased to background levels or as partial metabolic response (PMR) if residual uptake was observed. The patient characteristics of the collected data are shown in Table 1. Written informed consent for this clinical study was obtained from all the patients. This study was approved by [The Authors Institution] Institutional Review Board for Biomedical Research (approval no.: 3434).

**Table 1:** Baseline patient and tumor characteristics of the study cohort (*n* = 73)

	*n*
Age (median)	31–88 (59)
Tumor size (median)	1.9–11.2 (5.2)
FIGO 2018	
I	3
II	15
III	51
IV	4
Histology	
Squamous cell carcinoma	62
Adenocarcinoma	8
Small cell carcinoma	1
Glassy cell carcinoma	1
Adenosquamous carcinoma	1
Local recurrence	
Positive	10
Negative	63

### Imaging, treatment and follow-up

All patients underwent ^18^F-FDG-PET/CT and MRI within 1 month before RT initiation. Treatment consisted of concurrent CCRT. RTs included external beam RT and intracavitary brachytherapy for all patients, with boost irradiation administered to the lymph nodes in patients with metastasis. Among the 73 patients, 31 underwent three-dimensional image-guided brachytherapy (3D-IGBT), while the remaining 42 received conventional brachytherapy. Because only one recurrence occurred in the 3D-IGBT group, a stratified analysis based on the treatment modality was not feasible; therefore, all patients were analyzed as a single cohort. Most patients received cisplatin (CDDP) as a concurrent chemotherapy. A few patients received carboplatin (CBDCA), paclitaxel + CDDP, paclitaxel + CBDCA or bevacizumab. One patient did not receive chemotherapy.

For follow-up, MRI was performed within 1 month after the completion of RT. In contrast, PET/CT scans were scheduled between 2 and 3 months post-RT. This timing for PET was chosen because excessive inflammation typically subsides and clinical findings stabilize by 2 months—thereby reducing false-positive results—whereas the feasibility of salvage surgery diminishes if delayed beyond 3 months. Consequently, the mean interval between MRI and PET imaging was 52 days. Regarding clinical outcomes, all cases of recurrence were pathologically confirmed, with a mean interval from the completion of treatment to the diagnosis of recurrence of 291.1 days (range, 129–770 days).

### Magnetic resonance imaging acquisition and RECIST evaluation

The imaging sequences included T1-weighted imaging, T2-weighted imaging and diffusion-weighted imaging for pre- and post-treatment evaluations. Gadolinium-enhanced MRI was also performed in some patients. Tumor size was measured on MRI in accordance with the General Rules for Clinical and Pathological Studies of Uterine Cervical Cancer (4th Edition). The measurements were performed by two board-certified specialists: a radiologist and a radiation oncologist. Based on the post-treatment images, the patients were categorized into those showing a complete response (CR) and those showing a partial response according to the RECIST guidelines [13].

### 2-[^18^F]fluoro-2-deoxy-D-glucose positron emission tomography/computed tomography acquisition

^18^F-FDG-PET/CT imaging was performed using two different scanners at [The Authors Institution]: TOSHIBA Aquiduo (*n* = 44) and GE MEDICAL SYSTEMS Discovery 710 (*n* = 29).

Prior to ^18^F-FDG administration, the patients underwent a 6-h fast. Pre-scan measurements were performed as follows:

(1) Body weight: pretreatment, 38–91 kg (mean = 54.3 kg); post-treatment, 39–86 kg (mean = 53.2 kg)
(2) Blood glucose level: pre-treatment, 72–172 mg/dl (mean = 98.1 mg/dl); post-treatment, 71–156 mg/dl (mean = 96.6 mg/dl)

The dose of the radiopharmaceutical was determined based on the patient’s body weight and was administered as follows: pre-treatment, 109–317 MBq (mea*n* = 183.2 MBq); post-treatment, 114–290 MBq (mean = 180.6 MBq).

A quiet uptake period of ~60 min was maintained between administration and scanning: pre-treatment: 50–68 min (mean = 57.8 min); post-treatment: 51–70 min (mean = 58.2 min). The scanning process was concluded after an additional 60 min resting period.

Images were acquired from the vertex to the mid-thigh at a rate of 2 min per bed position. Attenuation correction was performed using an accompanying CT device. Image reconstruction was performed using the VUE Point FX method under the following conditions: filter cutoff = 4 mm, subset = 16, iteration = 2, and Sharp IR algorithm.

### ^18^F-FDG-PET/CT evaluation

All images were analyzed using a single software platform (AW server; GE Healthcare) under the supervision of a certified nuclear medicine physician (O.H.). SUV_max_, SUV_mean_, SUV_peak_, MTV and total lesion glycolysis (TLG) were measured for each pre- and post-treatment image [14–16].

For the region of interest (ROI) definition, the entire primary tumor was targeted in the pre-treatment images; for post-treatment images, the ROI was set to the residual uptake area if visible; if the lesion was not visually discernible, the ROI was defined as the anatomical region of the uterine cervix where the primary tumor was initially located. Local recurrence was defined as tumor regrowth at or adjacent to the site of the primary tumor. In all cases, post-treatment uptake was localized within the initial area of abnormal accumulation. Furthermore, the pathological diagnosis for all recurrent cases was identical to that of the pre-treatment tumor. To assess potential differences in metabolic response kinetics among histological subtypes, Student’s *t-*test was performed between the SCC group and other histological groups. No significant differences were observed, with two-tailed *P*-values for age, tumor size, post-treatment MTV, post-treatment SUV_max_ and post-treatment TLG being 0.686, 0.481, 0.483, 0.457 and 0.449, respectively. Thus, the absence of significant differences confirmed that histological subtypes did not act as a confounding factor in the subsequent metabolic response analysis.

(1) Standardized uptake value

The standardized uptake value (SUV) is a semiquantitative metric for PET images and is a widely used parameter. The SUV value is a dimensionless quantity calculated by normalizing the tissue radioactivity concentration (in Bq/ml) in the ROI to the administered dose per gram of body weight (Bq/g):


$$\mathrm{SUV}=\frac{\mathrm{Tissue}\ \mathrm{radioactivity}\ \mathrm{concentration}\ \left[\mathrm{Bq}/\mathrm{ml}\right]}{\frac{\mathrm{Injected}\ \mathrm{radioactivity}\ \mathrm{dose}\ \left[\mathrm{Bq}\right]}{\mathrm{Patient}\ \mathrm{weight}\ \left[\mathrm{g}\right]}}$$

SUV_max_ is the maximum uptake value within the ROI, and SUV_mean_ is the mean uptake value within the ROI. SUV_peak_ is defined as the mean uptake value within a 1 cm^3^ volume of interest centered at the voxel showing SUV_max_.

(2) Metabolic tumor volume

The MTV represents the volume of the region that demonstrates metabolic activity above a set threshold of the administered radiopharmaceutical. It is a parameter that reflects the tumor volume and incorporates the biological activity of cancer cells. For this measurement, a threshold SUV value must be set. Based on reports from the European Association of Nuclear Medicine (EANM) and prior studies, three different thresholds were used in this study, 21%, 31% and 41% of SUV_max_, and the MTV was measured for each [17–19].

(3) Total lesion glycolysis

TLG is a measure of overall lesion glucose metabolism and serves as an indicator for comprehensively assessing both tumor size and metabolic activity. It is calculated as the product of the MTV and SUV_mean_.

### PET Response Criteria in Solid Tumors

In this study, the metabolic response to CCRT was classified using PERCIST 1.0, in addition to the qualitative evaluation of ^18^F-FDG-PET/CT images [12].

The definitions for the PERCIST 1.0 are as follows:

(1) CMR: complete disappearance of FDG uptake in the target lesion, with reduced uptake to blood pool levels.
(2) PMR: A decrease in SUL_peak_ of ≥30% and by at least 0.8 SUL units between pre- and post-treatment scans.
(3) Stable metabolic disease (SMD): An increase or decrease in SUL_peak_ of <30%.
(4) Progressive metabolic disease: An increase in SUL_peak_ of ≥30% and by at least 0.8 SUL units in the target lesion, or the appearance of a new lesion.

However, because the anatomical location of the uterine cervix is close to the bladder, even cases with complete lesion disappearance might be judged as PMR because of physiological tracer excretion. Therefore, in this study, the uterine body after RT was used as the reference. A case was classified as CMR if the post-treatment SUV_peak_ of the uterine cervix was within the mean SUV_peak_ of the uterine body plus two standard deviations. Patients outside of this range were treated as having PMR or SMD. The mean SUV_peak_ of the uterine body was calculated for 71 patients, excluding two cases from the cohort of 73 patients owing to uptake or calcification within the uterine body. Two patients were excluded from the analysis: one with FDG uptake in the uterine body and one with calcification. Although the small sample size precluded a statistical comparison between these excluded cases and the study group, we measured their SUV_max_ values for reference. The SUV_max_ was 4.17 for the patient with uterine body uptake and 2.9 for the patient with calcification. Given that the mean SUV_max_ of the study group was 2.75, it is reasonable to infer that including the patient with calcification would have had a negligible impact on the overall analysis.

### Statistical analysis

Statistical analyses were performed using a single software package (SPSS Statistics version 28.0.1.0, International Business Machines Corporation, Armonk, New York, USA). A two-sided *P*-value of <0.05 was considered statistically significant.

The local control rate was analyzed as the primary endpoint to evaluate the prognostic significance of the clinical parameters. ROC analysis was performed to assess the accuracy of MTV at various thresholds for predicting local control, and the area under the curve (AUC) was calculated.

Furthermore, we performed ROC curve analysis to compare the predictive performance of the percent change in PET parameters with their absolute values before and after treatment. For the PET parameters derived from the most accurate MTV threshold and age at the start of treatment, ROC curve analysis was performed to determine the optimal cutoff values using the Youden index. Quantitative variables were dichotomized into two groups based on the calculated cutoff values.

Univariate analysis was performed using the log-rank test for dichotomized quantitative parameters, in addition to qualitative variables, such as size, Equivalent Dose in 2 Gy fractions, metabolic response, FIGO staging, pathology, RECIST evaluation and PERCIST evaluation. Here, metabolic response was defined by the subjective assessment of post-treatment images by the radiologist: CMR if no residual tumor was judged to be present and PMR if residual uptake was noted.

Finally, treatment response was evaluated using a combination of RECIST and PERCIST evaluations and a combination of RECIST evaluations and PET data. The sensitivity, specificity, positive predictive value (PPV) and negative predictive value (NPV) of these combined criteria were calculated. The PET data used in this study was the post-treatment MTV. For this combined assessment of treatment response:

(1) A result was classified as negative if the RECIST evaluation was CR or the PERCIST evaluation was CMR; otherwise, it was classified as positive.
(2) For the combination of RECIST and PET data, the result was considered negative if the RECIST evaluation indicated CR or if the PET data (post-treatment MTV) was below the cutoff value; otherwise, it was classified as positive.

## RESULTS

### Accuracy of MTV threshold

The ROC curves for the pre- and post-treatment MTV for local recurrence are shown in Fig. 1 using three different thresholds: 21, 31 and 41%. Although no significant difference was observed among the thresholds, Fig. 1B, which presents the post-treatment MTV ROC curve, indicated that the accuracy of the 21% threshold (AUC = 0.859) was superior. Therefore, a 21% threshold was adopted for all subsequent analyses. Furthermore, the AUC for all pre-RT ROC curves in Fig. 1A were < 0.7, whereas the AUC for all post-RT ROC curves in Fig. 1B was ≥0.7. These findings indicate that post-treatment PET parameters have better discriminatory ability for predicting local recurrence than pre-treatment parameters.

**Fig. 1:**
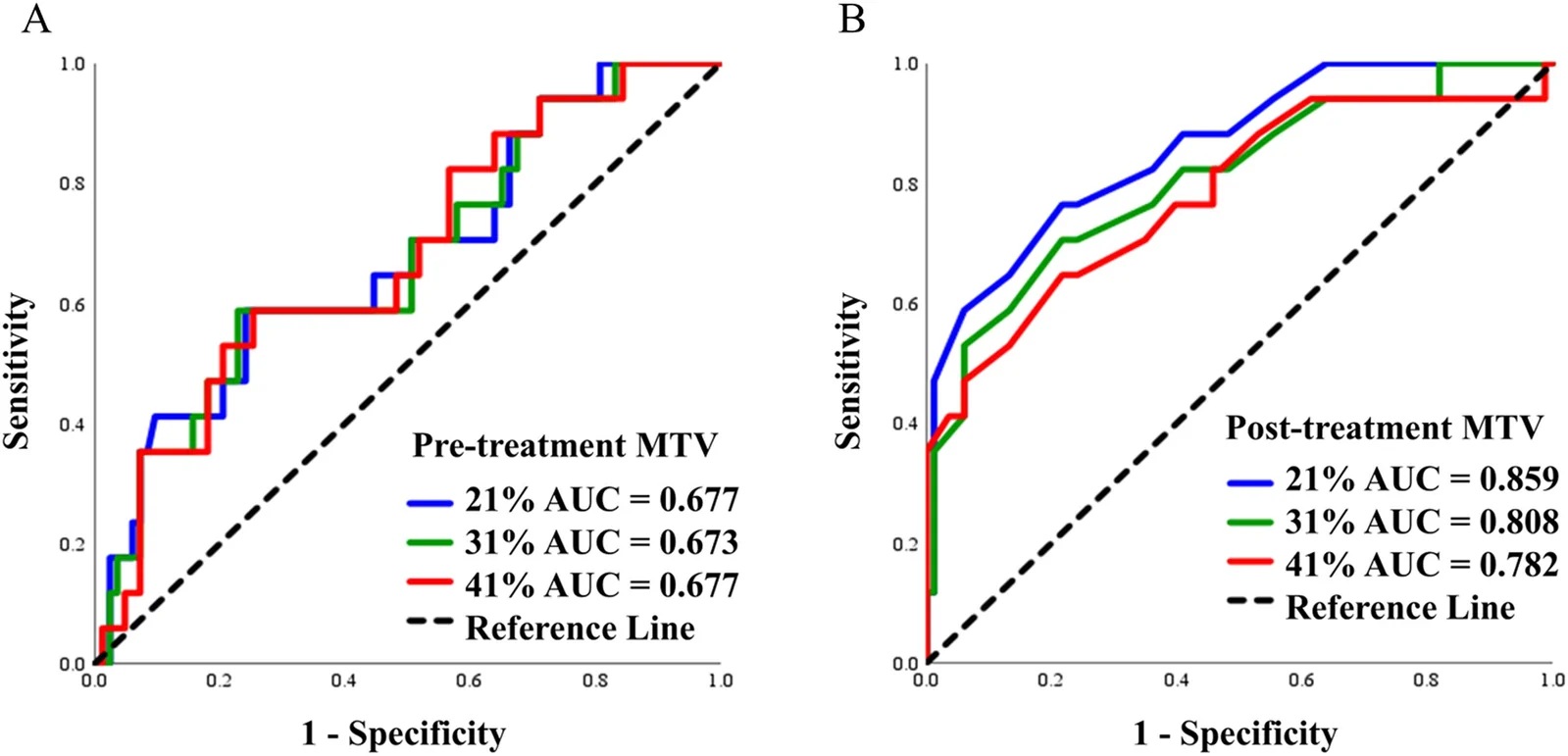
ROC curves A: Pre-RT MTV, B: Post-RT MTV.

### Receiver operating characteristic curve analysis

Figure 2 shows the ROC curves for predicting local recurrence using relative changes in PET parameters, while Fig. 3 presents the ROC curves using absolute values. The AUC values for the relative changes in Fig. 2 were low for all parameters (MTV = 0.489, SUV_max_ = 0.287, and TLG = 0.406). In contrast, the absolute post-treatment parameters in Fig. 3B exhibited high predictive accuracy. Because cervical cancer is highly radiosensitive, most cases showed a high rate of reduction, making it difficult to establish meaningful thresholds based on percentage changes. However, our findings indicate that the absolute value of residual uptake after treatment is a more significant predictor. Consequently, absolute values were adopted for all subsequent PET data analyses.

**Fig. 2:**
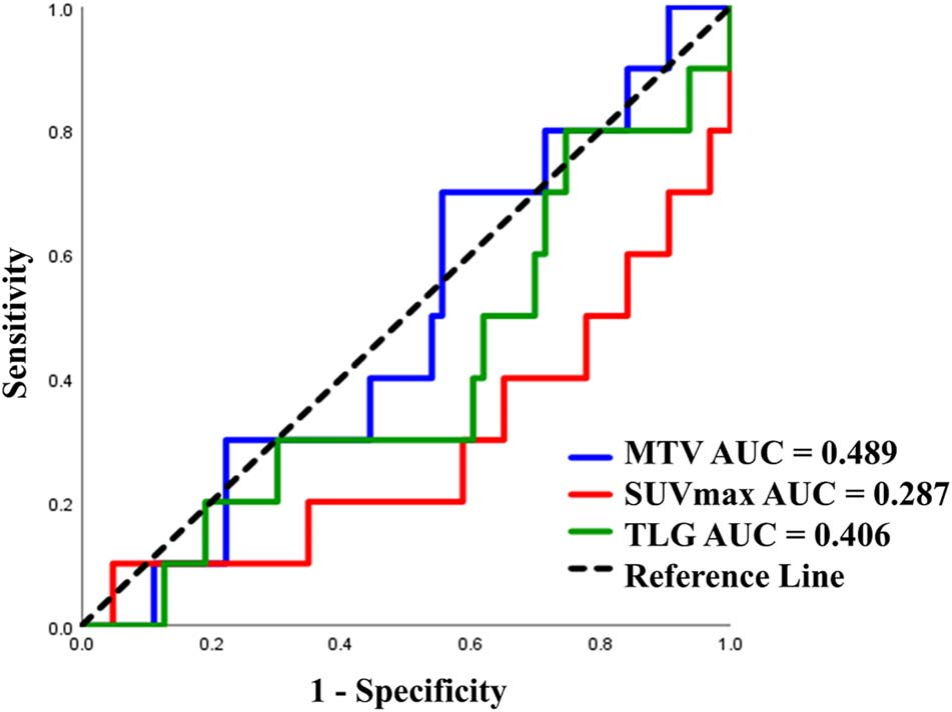
ROC analysis of the percent changes in PET data for predicting local recurrence.

**Fig. 3:**
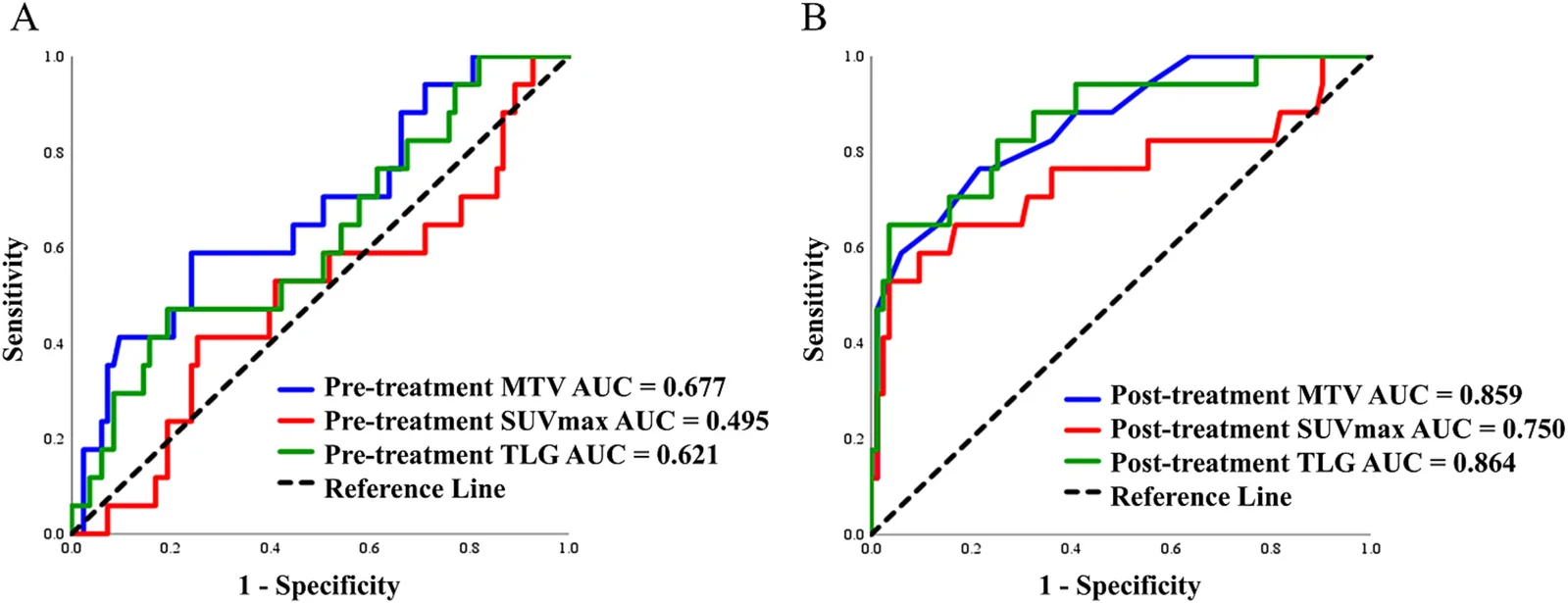
Pre- and post-RT ROC curves (A: Pre-RT, B: Post-RT).

Figure 3 compares the ROC curves for each PET parameter concerning local recurrence before and after treatment. It can be inferred that post-treatment PET parameters are more useful than pretreatment factors for predicting local recurrence (AUC: MTV = 0.859, SUV_max_ = 0.750, TLG = 0.864). Among these, the post-treatment TLG had the highest accuracy in this study.

Furthermore, the optimal cutoff values for each parameter, determined by the Youden index, were as follows: pre-treatment PET values: MTV = 73.25, SUV_max_ = 17.74, and TLG = 703.65; post-treatment PET values: MTV = 5.04, SUV_max_ = 4.17, and TLG = 15.05.

The sensitivity/specificity/PPV/NPV for predicting local recurrence regarding post-treatment MTV, SUV_max_ and TLG were as follows: MTV: 0.70/0.84/0.41/0.95, SUV_max_: 0.50/0.95/0.63/0.92 and TLG: 0.50/0.84/0.33/0.91.

These results indicate that the post-treatment PET parameters showed modest sensitivity but high specificity for predicting local recurrence.

### Univariate analysis

The results of the univariate analysis, obtained by classifying each parameter into two groups based on the optimal cutoff values calculated by ROC curve analysis, are shown in Table 2. Significant differences were observed in metabolic response (*P* = 0.035), pre-treatment MTV (*P* = 0.031), pre-treatment TLG (*P* = 0.014), post-treatment MTV (*P* < 0.001), post-treatment SUV_max_ (*P* < 0.001), post-treatment TLG (*P* < 0.001), and PERCIST evaluation (*P* = 0.013).

**Table 2:** Results of univariate analysis for predicting local recurrence

		*n*	Local control rate	*P* value
Age	<64.5	46	0.855	0.889
	≧64.5	27	0.830	
Tumor size	<4.0	17	0.933	0.267
	≧4.0	56	0.819	
FIGO 2018	I or II	39	0.827	0.668
	III or IV	34	0.865	
Histology	SCC	61	0.838	0.599
	Others	12	0.889	
EQD2	<85 Gy	64	0.824	0.213
	≧85 Gy	9	1.000	
RECIST	CR	62	0.852	0.549
	PR	11	0.818	
PERCIST	CMR	58	0.896	0.013
	PMR or SMD	15	0.667	
Metabolic response	CMR	45	0.923	0.035
	PMR	28	0.730	
Pre-treatment MTV	<73.3	50	0.910	0.031
	≧73.3	23	0.712	
Pre-treatment SUVmax	<17.7	54	0.853	0.765
	≧17.7	19	0.825	
Pre-treatment TLG	<703.6	57	0.902	0.014
	≧703.6	16	0.641	
Post-treatment MTV	<5.0	56	0.932	<0.001
	≧5.0	17	0.588	
Post-treatment SUVmax	<4.2	65	0.909	<0.001
	≧4.2	8	0.375	
Post-treatment TLG	<15.1	65	0.909	<0.001
	≧15.1	8	0.375	

SCC = squamous cell carcinoma, EQD2 = Equivalent Dose in 2 Gy fractions, CMR = complete metabolic response, PMR = partial metabolic response, SMD = stable metabolic disease

The Kaplan–Meier curves are presented in Fig. 4. Although the pre-treatment parameters for MTV were also significant, the post-treatment parameters yielded results with higher statistical significance. Additionally, there was a clear difference between the RECIST and PERCIST comparisons, indicating the high utility of ^18^F-FDG-PET/CT in predicting local recurrence after RT.

**Fig. 4:**
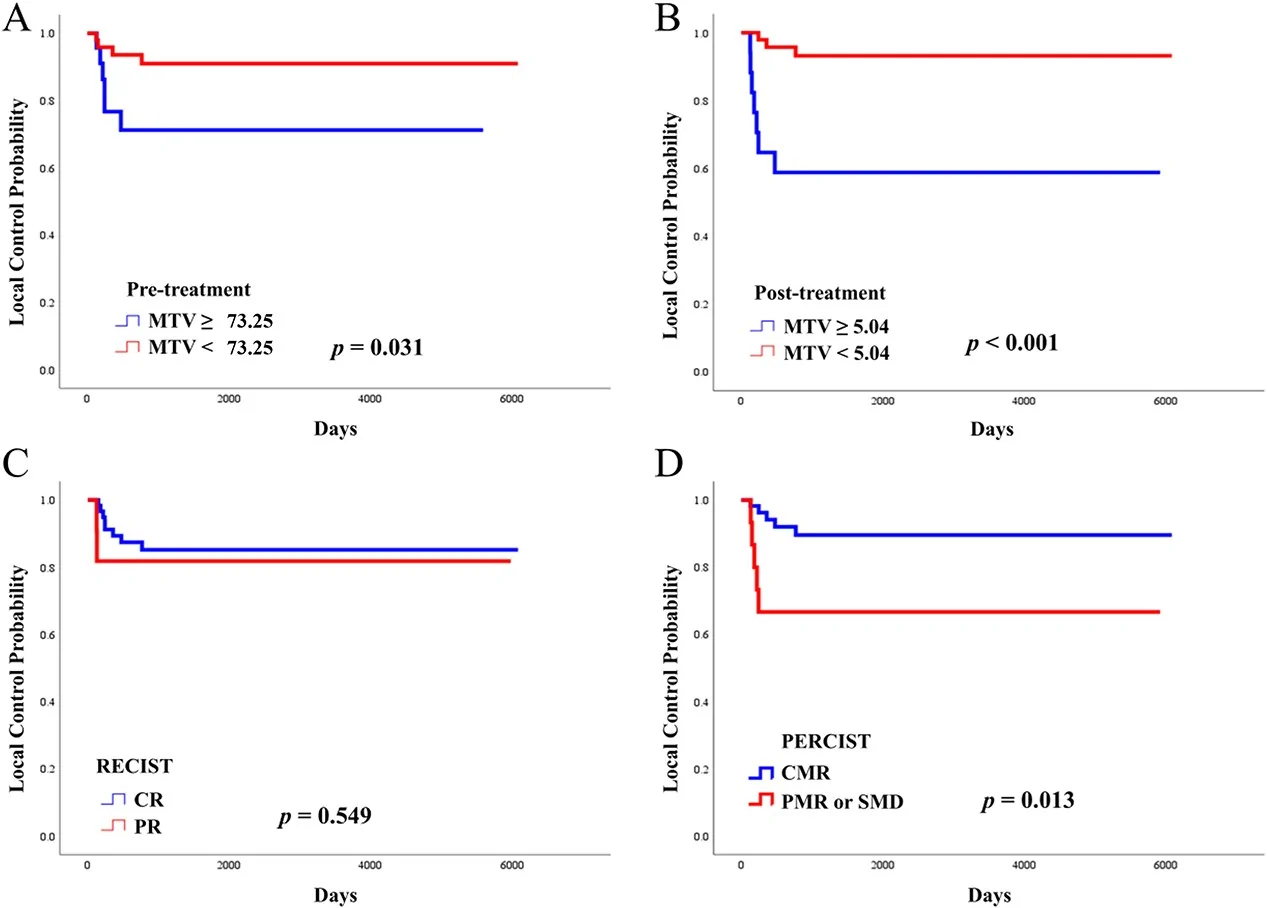
Kaplan–Meier curves A: Pre-RT MTV (*P* = 0.031), B: Post-RT MTV (*P* < 0.001). C: RECIST (*P* = 0.549), D: PERCIST (*P* = 0.013).

### Combined accuracy

The sensitivity, specificity, PPV and NPV of the combination of RECIST and PERCIST were 0.11, 0.97, 0.33 and 0.89, respectively. The sensitivity, specificity, PPV and NPV for the combination of RECIST evaluation and PET data were 0.10, 0.98, 0.50 and 0.87, respectively.

These results show that for both combinations, although the sensitivity and PPV are low, the specificity and NPV are high. This suggests that if the treatment effectiveness assessment yields a negative result, the possibility of recurrence is low and the patient can be managed with routine follow-up without the need for additional pharmacotherapy, thereby reducing the patient burden.

## DISCUSSION

### The utility of post-RT PET parameter measurement

Several previous studies have reported an association between pre-treatment PET data and prognosis, specifically overall survival (OS) and progression-free survival, in cervical cancer [20–23]. Similarly, in the present study, an association between pre-RT MTV and TLG and the prognosis for local recurrence was demonstrated. De Alencar *et al.* reported that the pre-RT SUV_peak_ showed the highest discrimination ability for predicting OS [24]. Conversely, Han *et al.* reported that patients with a high pre-RT MTV or TLG on PET had a higher risk of adverse events and mortality than those with low values [18]. Consistent with the report by Han *et al.*, the present study yielded significant results for the MTV and TLG. This difference can be attributed to the fact that the MTV and TLG are volume-based metabolic parameters that reflect the entire tumor, allowing for a more accurate prognostic evaluation.

Furthermore, reports have suggested that post-treatment PET data are superior to conventional imaging diagnostics for detecting recurrence and metastasis [25–27]. The present study yielded similar results and demonstrated that the post-RT PET data were more predictive than the pre-RT PET data. Chung *et al.* demonstrated that post-RT SUV_max_ could be used as a predictive indicator of recurrence [25]. Although concerning a different cancer site, Karaalioglu *et al.* reported that post-RT PET measurements of MTV and TLG were significantly associated with recurrence in nasopharyngeal carcinoma [26].

The presence of reports indicating the significance of SUV_max_ versus those indicating the significance of MTV or TLG is likely due to the difference mentioned earlier: MTV and TLG are volume-based and more accurately reflect the entire tumor, whereas SUV_max_ is simple to measure. In the present study, all post-RT parameters (SUV_max_, MTV and TLG) yielded significant results; however, SUV_max_ showed lower accuracy than the other parameters. Although MTV and TLG are highly accurate and useful for predicting local recurrence, they have drawbacks: the threshold used for MTV derivation varies among studies, and the calculation process is laborious. Conversely, SUV_max_ is less accurate but simpler to measure. Therefore, for clinical applications, the selection of an indicator should balance convenience with accuracy on a facility-by-facility basis.

### The optimal threshold for MTV delineation

Regarding the optimal threshold for MTV derivation, the present study found that a 21% threshold of SUV_max_ yielded the highest accuracy. Yu *et al.* reported that a threshold of 31 ± 11% demonstrated the best agreement for tumor volume contouring in non-small cell lung cancer [19]. Conversely, Ceriani *et al.* reported that a 25% SUV_max_ threshold was optimal for MTV calculations in primary mediastinal large B-cell lymphoma [28]. Furthermore, the EANM recommends a 41% threshold, which indicates that the optimal value widely varies across studies.

One possible factor contributing to the results of the present study is the influence of the adjacent organs. The bladder, an organ neighboring the cervix, physiologically shows high SUV, and this influence is likely to extend to the uterus. If the SUV_max_ of the cervix is affected by the physiological uptake in the bladder, a higher threshold may underestimate the primary tumor. Therefore, a lower threshold may delineate the primary tumor more accurately, allowing for more precise analysis.

In recent years, the number of reports using semiautomatic approaches, such as the iterative adaptive algorithm proposed by Xu *et al.* instead of fixed thresholds, has been increasing [29]. Given this background, it is essential to standardize the threshold for MTV calculations to ensure consistency in MTV measurements across institutions when MTV and TLG are used for treatment response assessments in the future.

### The utility of PERCIST as a treatment response assessment tool after RT for cervical cancer

In the comparison between the RECIST and PERCIST evaluations, the present study clearly demonstrated that treatment response assessment using the PERCIST evaluation was more accurate. Velez *et al.* reported that PERCIST 1.0 was superior in its ability to discriminate the OS rate among patients according to each evaluation criterion in metastatic castration-resistant prostate cancer [10]. Furthermore, Kim *et al.* reported that the overall response rate significantly increased when the European Organisation for Research and Treatment of Cancer criteria or PERCIST were adopted instead of the RECIST [30]. Additionally, Borcinova *et al.* reported that although the mean error between tumor size on MRI (used for RECIST evaluation) and tumor size on postoperative pathological examination was small, individual cases with large errors accounted for ~20% of the total, suggesting that MRI is not necessarily useful in all instances [8]. Similarly, the present study provides results supporting the utility of the PERCIST, suggesting that examinations using metabolic function assessments can detect changes in tumor viability earlier and are useful for prognostic prediction.

However, Zhao *et al.* pointed out that although evaluations using PERCIST showed the highest inter-reader agreement, its generalizability poses a challenge because of practical limitations in applying PERCIST in all cases [11]. The PERCIST has multiple eligibility criteria; in this study, ~30% of the total patient population was ineligible and excluded. Regarding the definition of assessment criteria, it is important to note that the original PERCIST thresholds were not directly applicable to all cases in this cohort. Consequently, we redefined the criteria for our analysis by specifically accounting for elevated FDG uptake in the background caused by the influence of adjacent organs. This highlights that the universal application of PERCIST is limited for cervical cancer; instead, the criteria must be re-evaluated and customized for each organ site. Therefore, generalizing PERCIST for cervical cancer requires careful consideration of organ-specific factors, necessitating further studies to achieve a broader consensus. Although the PERCIST is relatively inferior to RECIST in terms of practicality, the present study demonstrated that the PERCIST is useful for predicting prognosis by enabling the early detection of changes in tumor viability. Thus, the PERCIST is considered a valuable tool for assessing treatment response after RT.

### Evaluation of clinical application through combination of indicators

Finally, this study investigated the predictive accuracy by combining various indicators. Kishikawa *et al.* reported that combining the RECIST and PERCIST improved the PPV for head and neck SCC, and similar results were observed when combining RECIST and PET evaluations [9].

When similar analyses were performed in the present study, both combinations yielded high specificity and NPV. However, the sensitivity and PPV were low, and the specificity and NPV were similar to those obtained using post-RT PET data alone, indicating no additional benefit from combining the indicators.

This discrepancy with the results of previous studies is mainly attributed to the small number of patients in the present study, particularly the small number of recurrence cases, which precluded the accurate validation of sensitivity and PPV, and the difference in the cancer site.

Therefore, although this study did not demonstrate a clear added benefit from the combination of indicators, considering the utility shown in previous research and the fact that a certain number of cases are unsuitable for PERCIST application, the approach of combining RECIST evaluation and PET data holds promise as a prognostic management tool for cervical cancer. Further validation in a larger number of patients is warranted.

### Limitations

*This study has some limitations*.

First, this was a single-institution, retrospective study with a limited number of cases. In particular, while the transition from conventional brachytherapy to 3D-IGBT occurred during the study period, the number of local recurrence events in the 3D-IGBT group was extremely small (*n* = 1). This precluded a robust stratified analysis to account for potential confounding effects of evolving RT techniques. A large-scale accumulation of cases through multicenter collaborative research is warranted to validate the results and advance the discussion toward clinical application.

Second, the influence of adjacent organs on PET imaging may be a source of error. In cases where the cervix is adjacent to the bladder, there is a concern that difficulty in setting the volume of interest may lead to a decrease in analysis accuracy. In this study, contouring was performed by comparing PET and CT images. However, the influence of adjacent organs can be minimized by applying automated segmentation using artificial intelligence.

Third, the discrepancy in the timing of the PET and MRI scans is also a limitation. Post-RT PET scans were performed 2–3 months after the completion of treatment to avoid false positives caused by inflammatory reactions. In contrast, the timing of follow-up MRI was determined based on RECIST guidelines; therefore, aligning the schedules for both examinations was not feasible. This suggests that PET may be more reflective of the cumulative effect of RT, and thus, a simple comparison between the two modalities may be biased.

Fourth, immune checkpoint inhibitors (ICIs) were not utilized in this study. Given that ICI combination therapy is becoming the current standard of care, the predictive performance for recurrence at 3 months post-treatment may differ under these newer treatment regimens. It is important to emphasize that metabolic response alone should not be used as the sole basis for immediate decisions regarding salvage surgery or intensification of pharmacotherapy. Instead, we believe our findings should serve as a clinical indicator to prompt rigorous follow-up, including biopsy, to confirm the status of the lesion.

The final limitation concerns the definition of the PERCIST criteria. The original PERCIST guidelines define CMR as a reduction in the blood pool level, without being based on quantitative values. Therefore, if the research was conducted at multiple institutions, inconsistencies across facilities and analysts would be anticipated. In the present study, CMR was redefined based on the SUV of the normal uterine corpus to perform the analysis without subjective elements. However, if the PERCIST is used as a treatment response assessment tool after RT, consistency must be enhanced by standardizing the criteria for quantitative indicators.

Despite these limitations, few studies in Japan have investigated the utility of post-RT ^18^F-FDG-PET/CT and the validity of PERCIST evaluation in cervical cancer. The results of this study support the use of post-treatment ^18^F-FDG-PET/CT and represent an important finding that can contribute to improving local control rates for cervical cancer in Japan.

## CONCLUSION

Image analysis using ^18^F-FDG-PET/CT is useful for predicting residual and recurrent diseases in cervical cancer. Specifically, the SUV_max_, MTV and TLG derived from post-RT PET data are valuable. The findings suggest the high utility of PERCIST, which uses PET data, compared with the conventional treatment response assessment tool, RECIST evaluation.

## PRESENTATION AT A CONFERENCE

The 38th Annual Meeting of the Japanese Society for Radiation Oncology.

## CLINICAL TRIAL REGISTRATION NUMBER

The study design was approved by the Clinical Research Ethics Review Committee of Tokushima University Hospital (approval number: 3434).
